# Association of *DGAT1* and *POU1F1* Gene Polymorphisms With the Milk Traits in the Cross‐Bred Hamdani Sheep Bred Under Extensive Management

**DOI:** 10.1002/vms3.70565

**Published:** 2025-08-12

**Authors:** Ali Osman Turgut, Davut Koca, Mehmet Eroğlu

**Affiliations:** ^1^ Faculty of Veterinary Medicine Department of Animal Science Siirt University Siirt Türkiye; ^2^ Faculty of Veterinary Medicine Department of Obstetrics and Gynecology Van Yüzüncü Yil University Van Türkiye

**Keywords:** cross‐bred Hamdani, gene polymorphisms, milk composition, sheep

## Abstract

**Background:**

Milk production and composition are affected by genetic and environmental factors. Among key genetic regulators, the *POU1F1* and *DGAT1* genes play significant roles. *POU1F1* affects pituitary gland functions and hormone secretion, indirectly impacting milk production. *DGAT1* is crucial for milk fat synthesis. Understanding genetic variations in these genes can enhance breeding strategies for improved milk yield and quality.

**Objective:**

This study investigates the relationship between *POU1F1* and *DGAT1* genes and milk composition in cross‐bred Hamdani sheep.

**Methods:**

Blood and milk samples from 70 sheep were analysed for genetic markers using PCR‐RFLP technique. Statistical analysis assessed the relationship between genotypes and milk composition.

**Results:**

Results showed that *DGAT1* genotypes (CC and CT) were in Hardy–Weinberg Equilibrium (HWE), whereas *POU1F1* genotypes were not. C and T allele frequencies were found 0.9 and 0.1 for DGAT1, respectively. On the other hand, the frequencies of C and T alleles were 0.52 and 0.48 for *POU1F1*, respectively. For *DGAT1* gene, CT genotypes carrying ewes had higher milk fat compared to CC genotypes (*p* < 0.05). However, no differences were observed in milk solids‐not‐fat, protein and lactose content between genotypes (*p* > 0.05). Effect sizes were detected as 0.83 (large) for fat percentage comparison, 0.09 (small) for percentage of solids‐not‐fat and 0.06 (small) for percentage of protein and lactose. Association analysis did not perform for *POU1F1* gene due to low sample size in CC genotype.

**Conclusion:**

These findings suggest that *DGAT1* variations may influence milk fat content, whereas *POU1F1*’s role remains unclear due to limited genotype variation. Further research is needed to clarify genetic influences of *DGAT1* and *POU1F1* genes on sheep milk in cross‐bred Hamdani sheep.

## Graphical abstract

*DGAT1* gene AluI polymorphism affects milk composition in cross‐bred Hamdani sheep.

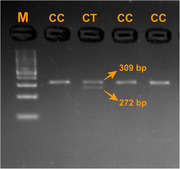

## 1 Introduction

Sheep have played a crucial role in human history for thousands of years, serving as a source of both meat and milk. The domestication of sheep dates back approximately 10,000 years, with early agricultural societies in Mesopotamia and Central Asia relying heavily on them for sustenance and economic stability (Zeder 1999; Alberto et al. 2018). Over the centuries, selective breeding has refined sheep breeds for various purposes, leading to significant variations in milk production. Today, sheep farming continues to be a major part of global agriculture, providing essential animal products worldwide (Haenlein and Wendorff 2006).

Milk is one of the fundamental components of human nutrition, offering a rich source of proteins, fats, vitamins and minerals (Turgut et al. 2023; Koca et al. 2023). Sheep milk has a higher fat and protein than cow milk, making it particularly valuable in the production dairy products (Turgut et al. 2023; Haenlein and Wendorff 2006). Enhancing sheep milk production and improving its nutritional quality is a critical goal for livestock farmers, dairy industries and consumers alike. The ability to maximize milk yield while maintaining desirable compositional attributes is essential for sustainable sheep farming (Turgut et al. 2023, 2024).

Milk yield and quality are determined by genetic and environmental factors. Among the genetic components influencing milk traits, *POU1F1* and *DGAT1* genes have been identified as key regulators in sheep (Mura et al. 2012; Tăbăran et al. 2014). The *POU1F1* (POU Class 1 Homeobox 1) gene regulates pituitary gland functions, indirectly affecting milk production through its influence on growth hormone, prolactin and thyroid‐stimulating hormone secretion (Bastos et al. 2006). Meanwhile, the *DGAT1* gene (diacylglycerol *O*‐acyltransferase 1) is critically involved in the synthesis of milk fat (Cases et al. 2001; Banilas et al. 2011). Understanding genetic variations within these genes and their impact on milk production could provide valuable insights into improving dairy sheep breeding programmes.

The cross‐bred Hamdani sheep, which are predominantly raised in Siirt province, Turkey, constitute the majority of the region's sheep population (Turgut et al. 2023; Işbilir et al. 2024; İşbilir and Güzel 2024). This breed is primarily utilized for both milk and meat production (Turgut et al. 2023), and its products play an important role in the preparation of traditional, animal‐based local foods (Gülmez et al. 2022). In addition to their nutritional value, Hamdani sheep also contribute significantly to wool and fur production, supporting local livelihoods and sustaining traditional crafts in the region (Gulaydin et al. 2025). However, limited information exists on gene polymorphisms related to milk traits in cross‐bred Hamdani sheep (Turgut et al. 2024). Considering this background, we aimed to investigate the relationship between *POU1F1* and *DGAT1* genes and some milk traits in cross‐bred Hamdani sheep. We hypothesize that specific genetic variations in these genes significantly affect milk composition, contributing to improved genetic selection strategies for enhancing sheep productivity.

## 2 Materials and Methods

### 2.1 Animals and Sampling

A total of 70 cross‐bred Hamdani sheep were used in the study. Age of ewes ranges between two and five. Ewes were fed with 0.3 kg barley and 0.5 kg lentil straw in addition to pasture. Ewes were fed in a barn that have good ventilation and reached to the water ad libitum. Before 10 h sample collection, lambs were separated from their mothers. Samples were collected from jugular vein into 6 mL K3‐EDTA containing tubes (Vacusera, Izmir, Türkiye). Milk samples were collected into 50 mL sterile falcon tubes from ewes during early lactation stages (between 30th and 40th days). Both blood and milk samples were transferred on the ice blocks immediately and stored at −20°C. Milk samples were analysed immediately using a milk autoanalyser (Lactoscan SA, Nova Zagora, Bulgaria). The results were also confirmed with reference laboratory methods described by Association of Official Analytical Chemists (AOAC). Moreover, the percentage of milk fat, protein, lactose and solids‐not‐fat (SNF), main component of milk, were determined. Genomic DNA was obtained using commercial DNA extraction kit (Hydra Biotechnology, Van, Türkiye). Integrity of genomic DNA was evaluated using both 1% agarose gel. Quality of genomic DNA was evaluated using a spectrophotometer (Allsheng, China). Optical density values (OD) of DNA samples were ranged between 1.7 and 1.9.

### 2.2 Polymerase Chain Reaction (PCR)

Primer pairs of *DGAT1* and *POU1F1* genes were presented in Table 1. Following PCR optimization, all PCR reaction were carried out on Kyratec SC300G thermal cycler (Kyratec, Queensland, Australia) in a 25 µL total volume as follow; 50 ng genomic DNA, EcoTaq 2X PCR mix (12.5 µL) (Songen Biotechnology, İstanbul, Türkiye), primers 5 pmol of each and nuclease free water up to final volume. PCR conditions were as follow; initial denaturation at 95°C—5 min, denaturation at 94°C (30 s), annealing at 60°C (30 s), extension at 72°C (30 s) and final extension at 72°C (7 min). Following PCR, amplicons were evaluated on the 2% agarose gel under UV using a gel imaging system (Gene‐Box, ER Biotech, Türkiye). Negative controls were used to evaluate PCR specificity.

**TABLE 1:** Primer pairs used in the polymerase chain reaction (PCR) amplification of *DGAT1* and *POU1F1*.

Gene	Primers (5′ → 3′)	PCR products (bp)	Reference
*DGAT1*	F: GCATGTTCCGCCCTCTGG R: GGAGTCCAACACCCCTGA	309	Yang et al. (2011)
*POU1F1*	F: GTATTGCTGCTAAAGACGCC R: GAGGGAAAGATATAGTGAAAGGG	469	Ozmen et al. (2014)

### 2.3 Restriction Fragment Length Polymorphism (RFLP)

PCR amplicons of both *DGAT1* and *POU1F1* gene regions were digested using *AluI* enzyme. RFLP reactions were performed at 37°C for 15 min in 50 µL total volume as follow; 10 µL PCR products, 10 U *AluI r*estriction enzyme, 10× rCutSmart Buffer (5 µL) and water up to final volume. Then, RFLP products of amplicons were loaded on 4% agarose gel at 120 V for 30 min. Then, genotypes were detected according to restriction pattern of products on the gel.

### 2.4 Statistical Analysis

Minitab (v21.4.1) package programme was used for analyses. Allele and genotypes frequencies were detected by direct counting. Hardy–Weinberg Equilibrium (HWE) was evaluated using chi‐square test. General linear model (GLM) was used to evaluate association of genotypes with the milk composition traits. Significance level was defined as *p* < 0.05. GPower (v3.1.9.7) was used to calculate effect size (Cohen's *d*) of pairwise comparisons of milk parameters. Significance level was defined as *p* < 0.05.

## 3 Results

**FIGURE 1:**
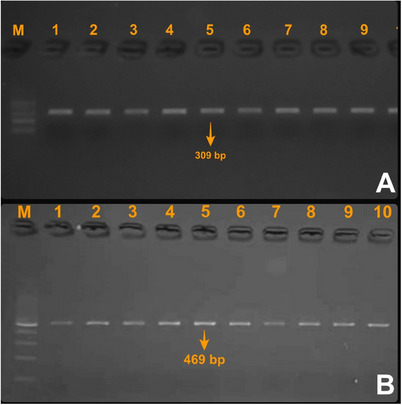
PCR products of *DGAT1* (A) and *POU1F1* (B) gene regions. M: 100 bp DNA ladder.

**FIGURE 2:**
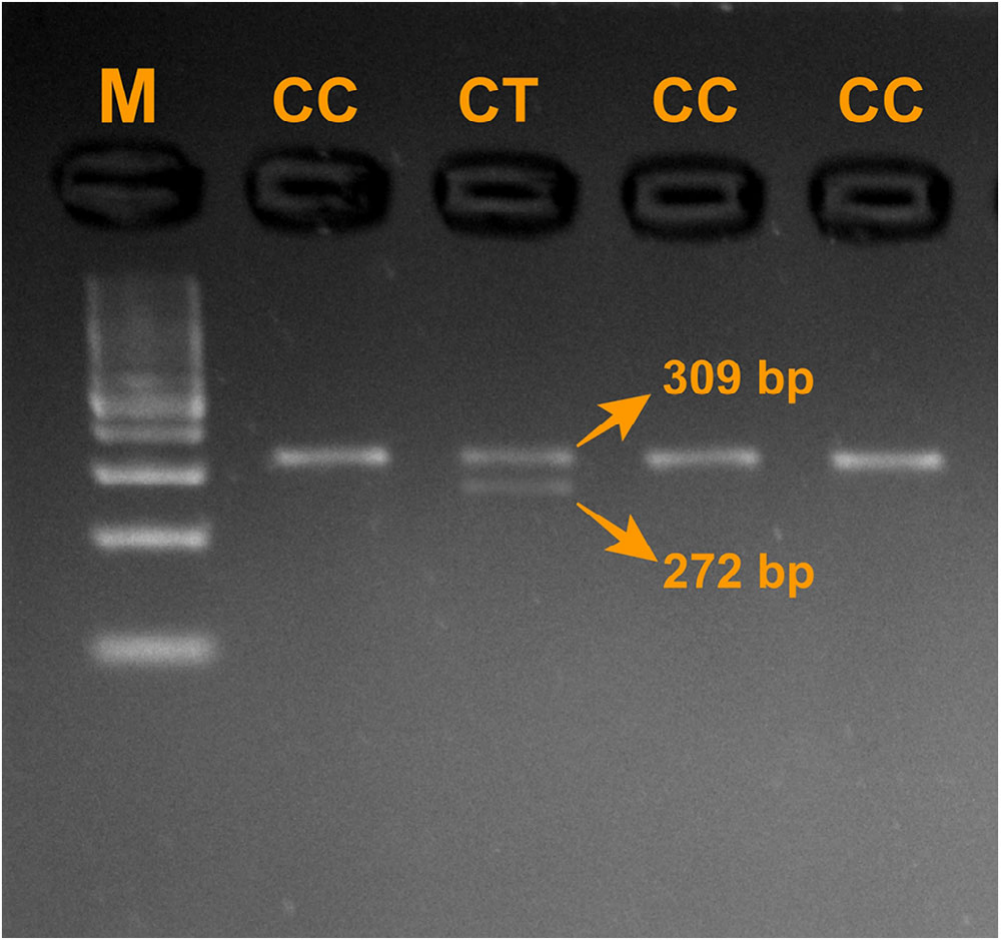
*AluI* RFLP pattern of *DGAT1*. M: 100 bp DNA ladder.

**FIGURE 3:**
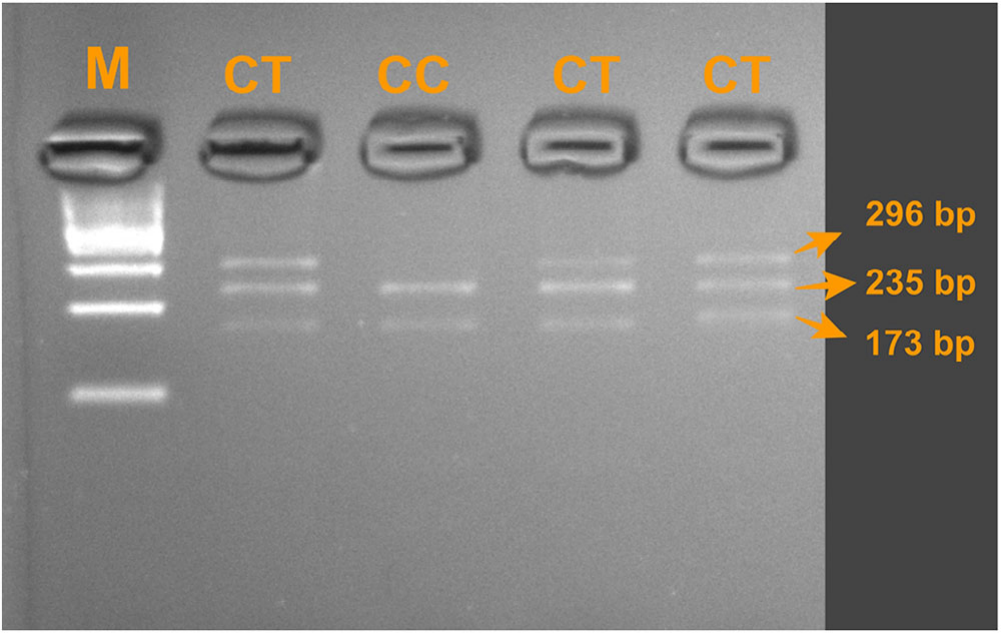
*AluI* RFLP pattern of *POU1F1*. M: 100 bp DNA ladder.

In the present study, a 309 bp PCR product of the *DGAT1* gene and a 469 bp PCR product of the *POU1F1* gene were successfully amplified in all analysed samples (Figure 1A,B). During RFLP analysis, the 309 bp *DGAT1* amplicons were digested using the *AluI* enzyme, allowing clear differentiation of genotypes. Two genotypes were identified: CC, represented by a single undigested 309 bp band, and CT, characterized by three fragments of 309, 272 and 37 bp (Figure 2). In contrast, digestion of *POU1F1* PCR products revealed different restriction patterns. The CC genotype showed bands of 235, 173 and 61 bp, whereas the CT genotype displayed bands of 296, 235, 173 and 61 bp (Figure 3).

Allele frequencies were calculated for both genes. For *DGAT1*, the C allele frequency was 0.90, and the T allele was 0.10. Conversely, for *POU1F1*, the C and T alleles were more evenly distributed, with frequencies of 0.52 and 0.48, respectively. Chi‐square analysis revealed that the *POU1F1* genotype frequencies deviated significantly from the HWE (*p* < 0.01), possibly indicating selection pressure or population structure. However, *DGAT1* genotypes were in equilibrium (*p* > 0.05) (Table 2). Due to the low number of ewes with the CC genotype (*n* = 3) for *POU1F1*, this locus was excluded from association analysis. For *DGAT1*, statistical comparisons revealed that ewes with the CT genotype had significantly higher milk fat percentages compared to those with the CC genotype (*p* < 0.05). No significant differences were observed in milk SNF, protein or lactose contents among *DGAT1* genotypes (Table 3).

**TABLE 2:** Allele and genotype frequencies of *DGAT1* and *POU1F1* genes.

		Allele frequency		Genotypes (observed)					
	*n*	C allele	T allele	CC	CT	TT	Ho	He	*p* value
*DGAT1*	70	0.9	0.1	56	14	—	0.200	0.180	>0.05
*POU1F1*	70	0.52	0.48	3	67	—	0.957	0.499	**0.005** ^**^

Abbreviations: HE, expected heterozygosity; Ho, observed heterozygosity.

** *p* < 0.01.

**TABLE 3:** The effects of *DGAT1* genotypes on milk composition (mean ± SE).

	*DGAT1* genotypes				
	CC	CT	TT	*p* value	Cohen's *d*
Fat (%)	7.043 ± 0.19	8.11 ± 0.42	—	**0.015** ^*^	**0.83**
Protein (%)	4.18 ± 0.04	4.16 ± 0.07	—	>0.05	0.09
Lactose (%)	3.95 ± 0.04	3.92 ± 0.06	—	>0.05	0.06
SNF (%)	8.80 ± 0.09	8.74 ± 0.15	—	>0.05	0.06

Abbreviation: SNF, solids‐not‐fat.

* *p* < 0.05.

Effect size estimation confirmed a large effect (0.83) for fat percentage. In contrast, small effect sizes were recorded for SNF (0.09) and protein and lactose (0.06), indicating that *DGAT1* variation mainly influenced milk fat content in this population.

## 4 Discussion

*DGAT1* and *POU1F1* genes some of the major genes affecting milk composition in the farm animals. In buffalo, polymorphisms in *DGAT1* gene were detected as a major gene associated with the milk fat and yield (Yuan et al. 2007; Mishra et al. 2007). Similarly, *DGAT1* gene polymorphism was associated with the milk production traits in various breeds in cattle (Tantia et al. 2006; Ardicli et al. 2018; Kęsek‐Woźniak et al. 2020). Polymorphisms in goat *DGAT1* gene were also related to milk production traits (An et al. 2013). In this study, polymorphism was detected in *DGAT1* gene for related SNP in Hamdani cross‐bred sheep. The same SNPs have been identified Chinese (Yang et al. 2011), Iranian (Mohammadi et al. 2013) and Turkish sheep breeds (Bayram et al. 2019) in previous studies. In Iranian sheep breeds, Moghani, Lori‐Bakhtiari sheep and Zel sheep, a SNP in *DGAT1* gene related to carcass traits (Mohammadi et al. 2013). In Turkish Akkaraman sheep, *DGAT1* gene polymorphisms have been related to birth weight (Bayram et al. 2019). However, limited studies investigated association of *DGAT1* gene polymorphisms with the milk composition (Tăbăran et al. 2014). In this study, as a first report of DGAT1, we detected CC and CT genotypes with higher C allele frequency (0.90) compared to T allele (0.10) contrary to Ozmen and Kul (2016). In addition, *DGAT1* genotype frequencies for related SNP were in HWE in cross‐bred Hamdani sheep. Furthermore, CT genotypes carrying ewes had higher milk fat compared to CC genotypes (*p* < 0.05). In addition, there was a large effect in the genotype comparisons for milk parameters. However, no differences were observed in the other composition traits between CC and CT genotypes (*p* > 0.05), and there were small effect sizes in the genotype comparisons.

Bastos et al. (2006) were the first to identify polymorphisms in the sheep *POU1F1* gene. Since then, limited studies were carried out for *POU1F1* gene and its relationship to milk traits in sheep. Mura et al. (2012) investigated *POU1F1* gene polymorphisms in Sarda sheep using PCR, SCCP and sequencing methods. They identified two novel SNPs: in the fourth intron (g.121C > T) and sixth exon (g.249G > A), which were not previously reported by Bastos et al. (2006). However, their statistical analysis found no significant relationship between these polymorphisms and milk traits. Bastos et al. (2006) had earlier identified substitutions in the *POU1F1* gene in exon 2, exon 3 and intron 4 in Churra da Terra Quente sheep. However, studies investigating *POU1F1* gene polymorphisms are limited in Turkish sheep breeds. In a study, Ozmen and Kul (2016) reported SNPs in exon 6 and the 3′UTR in *POU1F1* gene in Sakız sheep. A biallelic polymorphism was detected using the *AluI* enzyme, but no polymorphism was detected with *DdeI*. However, the *AluI* polymorphism was present with higher T (0.64) genotype frequency compared to C (0.64) genotype in Sakiz sheep. In addition, Ozmen and Kul reported significant relationship between genotype and milk traits in Sakiz sheep, where TT‐genotyped ewes had higher milk yield than CC‐genotyped ones, while CC individuals showed higher fat content. In addition, they reported many new SNPs in exon 6 and 3′UTR (Ozmen and Kul 2016). *POU1F1* gene polymorphisms were also related to milk traits and litter size in Iraqi Awassi sheep (Al‐Khuzai and Al‐Anbari 2018). In this study we have identified only CC and CT genotypes in cross‐bred Hamdani sheep with higher C (0.52) allele frequency compared to T allele (0.48). Furthermore, allele and genotype frequencies showed high heterozygosity in this breed with deviation from HWE. This genotype distribution shows the effect of selection or other factors in cross‐bred Hamdani sheep.

## 5 Conclusion

This study highlights the polymorphisms in *DGAT1* and *POU1F1* gene polymorphisms in cross‐bred Hamdani sheep. We found that both gene regions were polymorphic for related SNPs. For *DGAT1* gene, CT genotypes carrying ewes had higher milk fat compared to CC genotypes (*p* < 0.05). However, observed genotypes were not related to other milk composition traits in Hamdani cross‐bred sheep probably due to sample size. Therefore, more comprehensive studies should be carried out with bigger sample size in cross‐bred Hamdani sheep to improve milk composition and quality.

## Author Contributions

Ali Osman Turgut conceived the idea, arranged necessary funding and software, carried out experimental work and prepared original draft of the manuscript. Davut Koca and Mehmet Eroğlu prepared original draft of the manuscript, data analysis and preparation of original draft.

## Ethics Statement

This study was approved by Siirt University Animal Experiments Local Ethics Committee (Approval No: 2023/07/51).

## Conflicts of Interest

The authors declare no conflicts of interest.

## Data Availability

The data that support the findings of this study are available from the corresponding author upon reasonable request.
